# The Flour of the Family

**Published:** 1875-11

**Authors:** 


### THE FLOUR OF THE FAMILY.

The flour *of* the family, as well as *for* the family, is, we are inclined to think, the "Attrition Flour," which we spoke of last month. A friend tells us that Dr. Nichols, in the Boston *Journal of Chemistry*, has analyzed it, and commends it highly. We have not seen the number of the *Journal of Chemistry* containing his recommendation, as all our efforts to obtain a copy have resulted unsuccessfully; but we are prepared to acquiesce in Dr. Nichols' views in this regard. That the flour is a genuine, pure, whole-wheat product, appears quite certain from our investigations. We shall be glad to hear from all users of this new and apparently perfect product.

